# Vitamin D Status in Rheumatoid Arthritis: Inflammation, Arterial Stiffness and Circulating Progenitor Cell Number

**DOI:** 10.1371/journal.pone.0134602

**Published:** 2015-08-04

**Authors:** Alberto Lo Gullo, Giuseppe Mandraffino, Gianluca Bagnato, Caterina Oriana Aragona, Egidio Imbalzano, Angela D’Ascola, Francesco Rotondo, Antonella Cinquegrani, Enricomaria Mormina, Carlo Saitta, Antonio Giovanni Versace, Maria Adriana Sardo, Renato Lo Gullo, Saverio Loddo, Antonino Saitta

**Affiliations:** 1 Department of Clinical and Experimental Medicine, University of Messina, Messina, Italy; 2 Department of Biochemical, Physiological and Nutritional Sciences, University of Messina, Messina, Italy; 3 Department of General Surgery and Oncology, University of Messina, Messina, Italy; 4 Department of Biomedical Sciences and of Morphologic and Functional Images, University of Messina, Messina, Italy; University Hospital Medical Centre, GERMANY

## Abstract

**Background and Aims:**

Suboptimal vitamin D status was recently acknowledged as an independent predictor of cardiovascular diseases and all-cause mortality in several clinical settings, and its serum levels are commonly reduced in Rheumatoid Arthritis (RA). Patients affected by RA present accelerated atherosclerosis and increased cardiovascular morbidity and mortality with respect to the general population. In RA, it has been reported an impairment of the number and the activity of circulating proangiogenic haematopoietic cells (PHCs), including CD34+, that may play a role in endothelial homeostasis. The purpose of the study is to investigate the association between vitamin D levels and PHCs, inflammatory markers, and arterial stiffening in patients with RA.

**Methods and Results:**

CD34+ cells were isolated from 27 RA patients and 41 controls. Vitamin D levels, C-reactive protein (CRP), fibrinogen, pulse wave velocity (PWV), and carotid intima-media thickness (cIMT) were also evaluated. CD34+ count and vitamin D levels were lower in RA patients as compared to controls, while fibrinogen, CRP, PWV and cIMT were higher in RA patients. CD34+ cell number appeared to be associated with vitamin D levels, and negatively correlated to fibrinogen and early atherosclerosis markers (PWV and cIMT); vitamin D levels appear also to be inversely associated to fibrinogen.

**Conclusions:**

RA patients with moderate disease activity presented with low vitamin D levels, low CD34+ cell count, increased PWV and cIMT; we found that vitamin D deficiency is associated to CD34+ cell reduction in peripheral blood, and with fibrinogen levels. This suggests that vitamin D might contribute to endothelial homeostasis in patients with RA.

## Introduction

Rheumatoid arthritis (RA) is a systemic inflammatory disease associated with increased morbidity and mortality, mainly due to cardiovascular events [1]. The mechanisms behind the higher prevalence of cardiovascular disease (CVD) in RA is not fully understood, but it is likely that the incidence may be linked to vascular inflammation and consequently to accelerated atherosclerosis [2, 3]. Atherosclerosis and RA share many common inflammatory mediators, and the mechanisms leading to synovial inflammation are similar to those found in unstable atherosclerotic plaque; levels of inflammatory molecules, such as CRP, fibrinogen and cytokines, including interleukins (IL-s), may be altered in RA and promote proatherogenic activation and endothelial dysfunction, but also associate with other CV risk factors, such as changes in lipid levels, insulin resistance and oxidative stress that further contribute to vascular damage [2–4]. Circulating proangiogenic hematopoietic cells (PHCs) are a heterogeneous population of cells in different states of maturation with the ability to differentiate into a broad range of cell types of different organs and systems, including cardiomyocytes, smooth muscle cells, and endothelial progenitor cells, as well as hematopoietic, stromal, and epithelial cells [5–7]. However, PHCs have been shown to contribute to postnatal vasculogenesis and they may participate in the turnover of healthy and damaged endothelium, delaying the development of atherosclerosis and CVD [6]. PHCs are negatively affected by risk factors for CVD and positively by changes in lifestyle, are associated with life expectancy, and their number is considered an independent predictor of CVD and all causes mortality [6, 8, 9].

Many different surface antigen, often co-expressed by endothelial and hematopoietic cells, have been already proposed to identify putative endothelial progenitors cells (EPCs), including CD34, CD117, CD133, CD105, CD144, CD184, CD309 (KDR or VEGFR2), acetylated low density lipoprotein, and various plant lectins [7]; however, the question of which cell phenotype better identifies the “true” circulating EPC remains unsolved, since the more widely studied PHC phenotypes do not give rise to mature ECs and are different from endothelial forming colony cells [10, 11]. Despite consistent evidence of CD34 expression by many different cell types, there is still a misconception that this surface antigen identifies a cell of hematopoietic origin, and experimentally, CD34+ cells are often regarded as hematopoietic contamination and subsequently disregarded [6]. Although the mechanisms on how CD34+ cells exert their role in angiogenesis are uncertain CD34 appears to identify a cluster of cell types with progenitor and stem activity, and in many cases, the CD34+ population showed a more potent or pronounced differentiation capacity, and also transdifferentiation ability [6]. In addition, it was suggested that mature endothelial cells may de-differentiate and re-establish an overlapping endothelial-hematopoietic phenotype, including CD45 expression, also in adulthood; thus, even the expression of CD45, which is generally considered a specific pan-leukocyte marker, might not be a reliable watershed between the hematopoietic and endothelial lineage [12, 13]. Fadini and coll. evaluated the impact of different immunophenotypes of CPCs on the ability to predict or associate with CV risk factors and outcomes. They suggested that CD34+ cell count is closely linked to CV risk, better than CD133+ cell number and multiple positive phenotypes (CD34+/CD133+, CD34+/KDR+, CD133+/KDR+, and triple positive CD34+/CD133+/KDR+), and an extensive antigenic characterization of circulating CD34+ cells may not be useful for this purpose [14, 15]. Consistently, CD34+ cell count has been suggested for CVD risk estimation in the clinical practice, rather than more complex EPC phenotyping, since the quantification of CD34+ cells, although not-specific for endothelial/CV repair, is already performed in most hematology laboratories in a standardized fashion [5, 6, 12]. Furthermore, a recent article indicated that CD34 hybrid cells promote endothelial colony-forming cell bioactivity and have a therapeutic potential for ischemic diseases [16]. Indeed, although it has been suggested that in RA patients the impairment of endothelial repair mechanisms may be at least in part due to the altered number or function of PHCs, many other cell types may cooperate in maintaining endothelial homeostasis, including the so-called angiogenic T cells (Tang), a subset of T cells recently described; Tang, in fact, seems to cooperate with circulating progenitors and enhance endothelial repair function, possibly through the secretion of proangiogenic cytokines [16–18].

In RA, PHCs are recruited into synovia and may participate in local angiogenesis [19]; the consequence of PHCs migration to the inflamed synovia would be paralleled by a reduction in the periphery [20] potentially impairing endothelial repair and leading to vascular damage. However, additional factors could be involved in modulating the number and activity of progenitor cells in RA [21, 22].

Adequate vitamin D status is important for optimal function of many organs and tissues throughout the body, including the cardiovascular system [23]. In several studies Vitamin D deficiency has been associated with atherosclerosis and cardiometabolic risk factors in non-RA populations, such as metabolic syndrome, hypertension, insulin resistance and CVD, as arterial dysfunction, arterial stiffness (AS), and atherosclerotic coronary disease [23–26]. In RA, low levels of vitamin D are noted to be common [27] and are described to be associated with different cardiometabolic risk factors [28].

Recently, vitamin D receptor has been detected in PHCs [29]. Furthermore, it has been described that vitamin D3 promotes cell proliferation of endothelial colony-forming cells by increasing vascular endothelial growth factor (VEGF) expression and pro-matrix metalloproteinases (MMP) activity [30]. Those data support the hypothesis that vitamin D might have a role in endothelium homeostasis.

The relationships between vitamin D, inflammatory markers, AS and PHCs in RA patients have not to date yet been investigated. An observational cross sectional study was designed to investigate the association between CD34+ cell number, vitamin D levels, and systemic inflammation. Pulse wave velocity and carotid intima-media thickness (cIMT), both patterns of preclinical atherosclerosis, were also evaluated in relation to inflammatory markers, CD34+ cell number, and vitamin D levels.

## Materials and Methods

### Subjects

**Fig 1:**
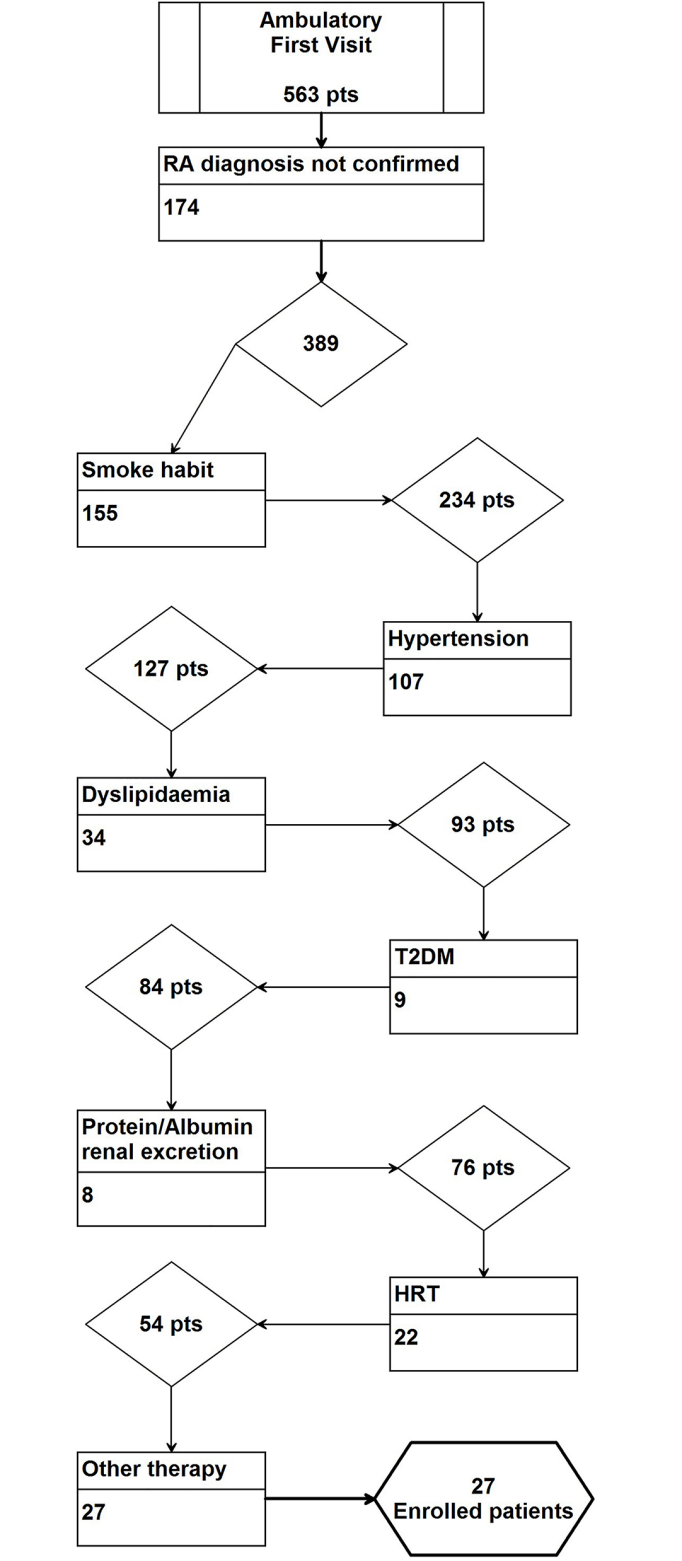
Flow diagram for patients exclusion.

Between October 2012 and May 2013, 563 outpatients were examined for the first time at the Rheumatology Division of the University of Messina and were referred for a clinical and instrumental screening; according to inclusion/exclusion criteria, only 27 subjects (8 men and 19 women) were considered eligible for this study (Fig 1): to be recruited for the study, subjects needed to be newly diagnosed, untreated, not have additional risk factors for atherosclerosis or CVD, and meet the retrospective application of the 1987 revised RA criteria of the American Rheumatism Association [31]; additionally, they should never have been treated with immunosuppressive drugs, long term corticosteroids and/or NSAIDs nor DMARDs. Patients previously treated with NSAIDs should not take drugs for at least two weeks before inclusion. To select a homogeneous population, only patients with “moderate” disease activity were enrolled, according to DAS28 value; DAS28 was calculated using CRP and the Nijmegen formula: DAS28 (CRP) = 0.56*(TJC28)^1/2^ + 0.28*(SJC28)^1/2^ + 0.014*GH + 0.70*ln (CRP). Subjects with co-morbidities, such as diabetes mellitus (T2DM), dyslipidemia (defined as plasma levels of cholesterol ≥230 mg/dl or low-density lipoprotein cholesterol (LDL-C) ≥160 mg/dl, or triglycerides ≥250 mg/dl), hypertension (defined as systolic blood pressure (SBP) ≥140 mmHg and/or diastolic blood pressure (DBP) ≥90 mmHg) were excluded from the study. Smokers and patients previously supplemented with vitamin D analogues or derivates were also excluded. Women taking hormone-based therapy were not included in the study. Thyroid, liver or kidney diseases, body mass index (BMI) ≥30, alcohol consumption, abnormal electrocardiographic or echocardiographic (Left Ventricular Ejection Function, Left Ventricular Regional Function) pattern, and clinical history of CVD were also considered as exclusion criteria. To reduce the influence caused by seasonal variations in vitamin D status throughout the year, the participants were enrolled between October and May. Systematic pharmacological therapy was started after clinical and instrumental examinations.

Forty-one subjects (13 men and 28 women) matched for age and gender were enrolled from hospital personnel as control subjects. After inclusion in the study, patients and controls underwent blood sampling and instrumental examination as described below; patients were then referred to the rheumatology clinic for clinical and therapeutic follow-up.

### Ethics Statement

Written informed consent was obtained from all subjects according to the Helsinki declaration and the observation was approved by the Ethics Committee of the University of Messina.

### Methods

All chemical analyses were performed at the medical centre after an overnight fasting. Plasma lipids, glucose, fibrinogen, rheumatoid factor (RF) were determined by routine methods. CRP, anti-cyclic citrullinated peptide antibodies (aCCP) were determined by a commercially available ELISA kit.

25-hydroxyvitamin D3 (25-OH D) was measured using high-performance liquid chromatography (Bio-Rad Laboratories). According to Ross et al, Vitamin D deficiency was defined as 25-OH D levels <20 ng/ml, insufficiency as 25-OH D levels between 20 and 30 ng/ml and optimal levels as 25-OH D levels >30 ng/ml [32].

### CD34+ cells identification and count

**Fig 2:**
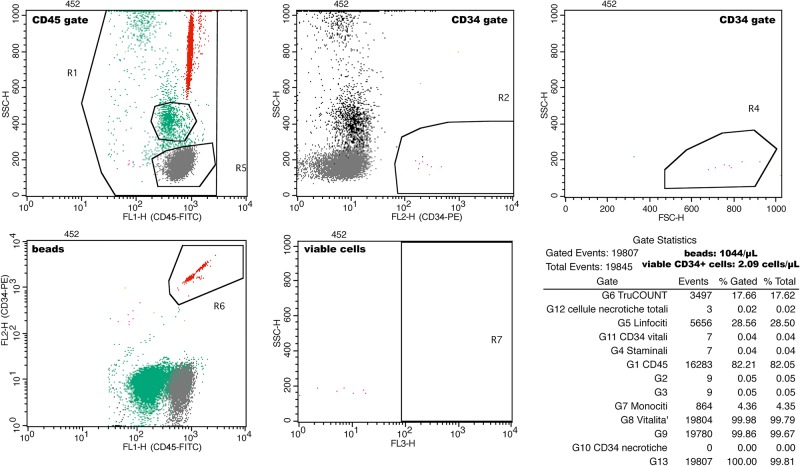
Flow citometry sequential gating strategy.

Flow cytometry (FACSCalibur; Becton Dickinson and Co., Franklin Lakes, NJ, USA) was used for cell identification. For this study, we identified and counted circulating CD34+ cells in peripheral blood. The cells were analyzed for the expression of surface antigens with direct multi-color analysis using fluorescein isothiocyanate (FITC)-conjugated, and phycoerythrin (PE)-conjugated monoclonal antibodies (mAbs) by flow cytometry analysis, as reported elsewhere [33]. Staining and analysis were performed using the International Society of Hematotherapy and Graft Engineering (ISHAGE) sequential strategy [34]. All peripheral blood samples were collected and stored in 0.34 M K3EDTA anticoagulant and analyzed within 2 h. Fifty μL of peripheral blood was then incubated with 10 μL of FITC-conjugated anti-human CD45 mAb (Becton-Dickinson, BD, San Jose, CA, USA) and with 10 μL PE-conjugated anti-human CD34 mAb (BD), using the multiparameter flow cytometric lyse no-wash method PROCOUNT (BD) in a TRUCOUNT tube (BD) with a known number of fluorescent beads (as reported on label of each BD TRUCOUNT lot); BD Pharm Lyse was used as Lysing Buffer. Incubation was performed at room temperature for 15 min in the dark. Next, 7-amino-actinomycin D (7-AAD; VIA-PROBE, BD Pharmingen, San Diego, CA, USA) was added to identify dead cells. To avoid cell loss, no wash was performed. Flow cytometric acquisition and analysis were performed by FACSCalibur; the threshold was set on FITC fluorescence in a dot plot of CD45-FITC vs side scatter (SSC) to exclude debris and ensure that all leukocyte populations and microbeads were included. Gating strategies and sample analyses allowed the identification of CD34+ cells by using the Macintosh CELLQuest software program (BD). Cell number was expressed as absolute count following manufacturer instructions, as reported below; during analysis, the absolute number (cells/μL) of positive cells in the sample can be determined by comparing cellular events to beads events, according to the formula: cell population absolute count = (number of event in cell populations/number of events in absolute count bead region)*(number of bead-test/test volume) (Fig 2). Only viable cells were counted; dead cells were excluded due to the use of 7-AAD. Consistenty, viable CD34+ cells were identified and counted.

### Measurement of cIMT and AS indices

Carotid echo Doppler scan and AS assessment were performed using a Vivid 3 Expert ultrasound machine equipped with a 7–15 MHz linear array transducer (GE Healthcare, Horten, Norway). cIMT has been expressed as the mean value of six-points IMT (common carotid, bifurcation, and internal carotid, bilaterally); according to ESC/ESH guidelines, we considered as carotid wall thickening a cIMT ≥0.9 mm [35], and carotid plaque ≥1.3 mm. PWV was determined by acquiring waveforms at the carotid and femoral arterial sites with electrocardiogram gating. Velocity (distance/time in m/s) was calculated by measuring the time interval between electrocardiogram R-wave and the recorded waveforms at each site, whereas distance between sites was measured manually.

The instrumental evaluations were repeated twice in duplicate by two experienced independent operators. The intraobserver/interobserver variability of IMT measurements was 0.01/0.02 mm; intraobserver/interobserver variability of PWV was 6.1/8.2%.

### Statistical analysis

The Kolmogorov-Smirnov test verified that several variables had a non-normal distribution; consequently, we chose a traditional non-parametric approach (median and IQR) and, consistently, the variables were compared using the Mann-Whitney test. To increase the power of statistics, given also the small size of our sample, we chose to use the non-parametric combination test (NPCT), based on a permutation solution within a resampling procedure, as already suggested elsewhere [36]. Consistently, data are also shown as median ± standard deviation (SD). The correlations among the variables were assessed by Spearman’s test. To assess the contribution of each variable on study variables a linear, stepwise, multivariate regression analysis was performed that allows considering continuous and categorical variables together on the whole study population. A two-tailed alpha of 0.05 was used to denote statistical significance. SPSS statistical package (ver. 17.0, Chicago, IL), and NPC test 2.0 –Statistical software for multivariate permutation tests (Methodologica srl, Treviso, Italy) were used to perform statistical analyses.

## Results

**Fig 3:**
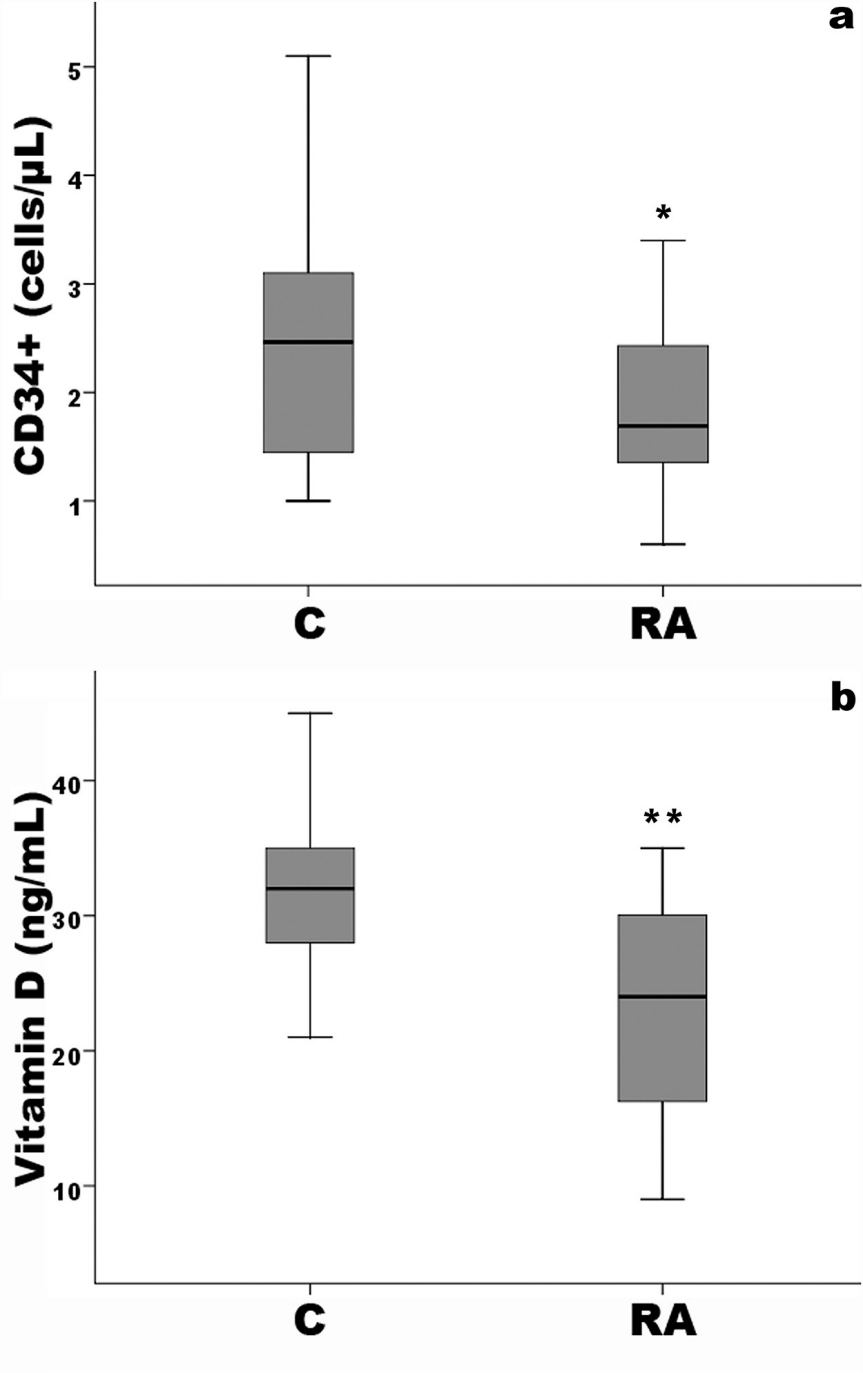
Box and whiskers plots for CD34+ cell number (a) and Vitamin D levels (b) in controls (C) and RA patients (RA). *p<0.01 vs controls, **p<0.001 vs controls. Solid horizontal lines = median values; error bars = 95% Confidence intervals; Shaded area = Interquartile range.

**Fig 4:**
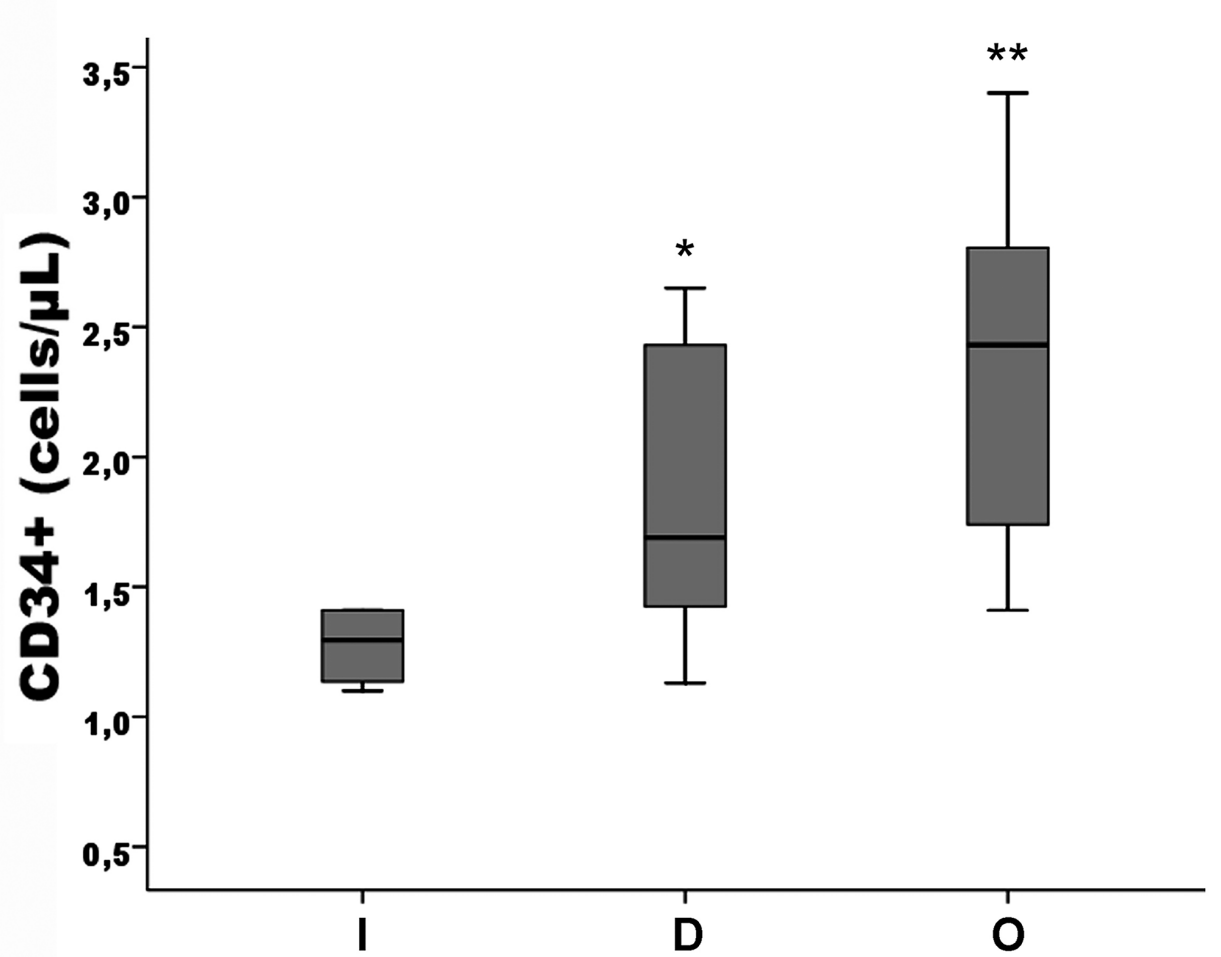
Box and whiskers plots for CD34+ cell number in RA patients with insufficient (I), deficient (D), and optimal (O) vitamin D levels. *p<0.01 vs controls, **p<0.001 vs controls. Solid horizontal lines = median values; error bars = 95% Confidence intervals; Shaded area = Interquartile range.

Table 1 shows the baseline characteristics of the study groups. No difference was detected as regards age, BMI, gender, blood pressure, glucose, lipid profile. Fibrinogen, PWV and also cIMT values was significantly higher in RA patients compared to controls (p<0.001) as was CRP (p<0.05); moreover, cIMT was on average above 0.9 mm, considered the upper reference limit for preclinical atherosclerosis according to ESH-ESC guidelines [35]. Vitamin D levels and CD34+ cells were significant lower respect to controls (both p<0.001). The disease activity score (DAS) and the duration of disease are also reported in Table 1. Eighteen of 27 patients with RA were both RF and aCCP positive, nine were only RF positive. In RA, vitamin D insufficiency was found in 10 patients, vitamin D deficiency in 7 patients and sufficiency levels of vitamin D in 10 patients. Fig 3 shows box and whiskers plots for CD34+ cell number (a) and vitamin D levels (b), in controls and RA patients. Fig 4 shows box and whiskers plots for CD34+ cell number in RA patients according to have vitamin D deficiency (D), insufficiency (I) or optimal plasma levels (O).

**Table 1:** Characteristics of study population.

	RA		CONTROLS			
Number	27		41			
Gender (m/f)	8/19		13/28			
	Mean±SD	Median (IQR)	Mean±SD	Median (IQR)	NPCT	Mann-Whitney
Age (years)	47.5±12.5	47 (12)	46.4±4.1	46 (3)	0.474	0.577
BMI (kg/m^2^)	25.2±4.2	25 (6.2)	23.8±2.5	23.5 (3.6)	0.12	0.08
SBP (mmHg)	125.2±10.3	125 (15)	121.6±9.1	120 (18)	0.13	0.16
DBP (mmHg)	73.1±11.9	70 (15)	70.7±6.3	70 (10)	0.15	0.12
TC (mg/dl)	191.9±43	192 (75)	181.9±21	180 (36.5)	0.08	0.21
HDL-C (mg/dl)	63.5±14.9	61 (20)	54.5±10.3	53 (9)	0.002	0.004
TG (mg/dl)	109.9±60.8	81 (67)	108±20.8	103 (22.5)	0.8	0.11
LDL-C (mg/dl)	109.5±40.8	100 (61.6)	108.1±32.5	108.2 (37.8)	0.8	0.8
Glucose (mg/l)	94.5±17.3	94 (16)	88.2±6.7	88 (11)	0.08	0.12
Hs-CRP (mg/dl)	0.9±1.1	0.65 (1.1)	0.4±0.22	0.4 (0.3)	<0.001	<0.05
Fibrinogen (mg/dl)	336.6±61	325 (81)	271.9±56	291 (95.5)	<0.001	<0.001
DAS 28	4.1±0.75	3.8 (1.37)	-	-	-	-
Duration disease (months)	5.1±3.9	4 (3)	-	-	-	-
25-OHD (ng/ml)	23±7.6	24 (15.1)	31.7±5.2	32 (7.20)	<0.001	<0.001
CD34+ *(cells/μL)*	1.8±0.6	1.7 (1.20)	2.5±0.9	2.46 (1.85)	0.0010	<0.01
PWV (m/s)	8.38±2.43	7.55 (4.22)	4.73±0.74	4.95 (0.75)	<0.001	<0.001
cIMT (mm)	1±0.17	1.1 (0.25)	0.76±0.13	0.71 (0.2)	<0.001	<0.001

Values are median (IQR) or mean ± SD. Abbreviations: BMI: body mass index; SBP: systolic blood pressure; DBP: diastolic blood pressure; TC: total cholesterol; HDL-C: high density lipoprotein-cholesterol; TG: triglycerides; LDL-C: low density lipoprotein cholesterol; Hs-CRP: high sensitivity C-reactive protein.; DAS 28: Disease Activity Score; PWV: pulse wave velocity; cIMT: carotid intima-media thickness. p: p value level for two tailed Mann-Whitney test or NPCT, RA patients versus controls.

We verified whether any correlation exists between CD34+ cell number, inflammatory markers (CRP, Fibrinogen), 25-OH D, and PWV in RA patients.

In RA patients we found a correlation between CD34+ cell number and vitamin D (rs = 0.706 p<0.001), fibrinogen (rs = -0.546, p<0.001), PWV (rs = 0.517, p<0.01) and cIMT (rs = 0.451, p<0.05), but not with CRP, DAS, or blood pressure values. A correlation was found between vitamin D and fibrinogen (rs -0.491, p<0.01), and with CD34+ cell number (rs 0.706, p<0.001), but no correlation was found between vitamin D serum levels and CRP, PWV, cIMT, lipids, glucose, blood pressure, RF, or aCCP.

**Fig 5:**
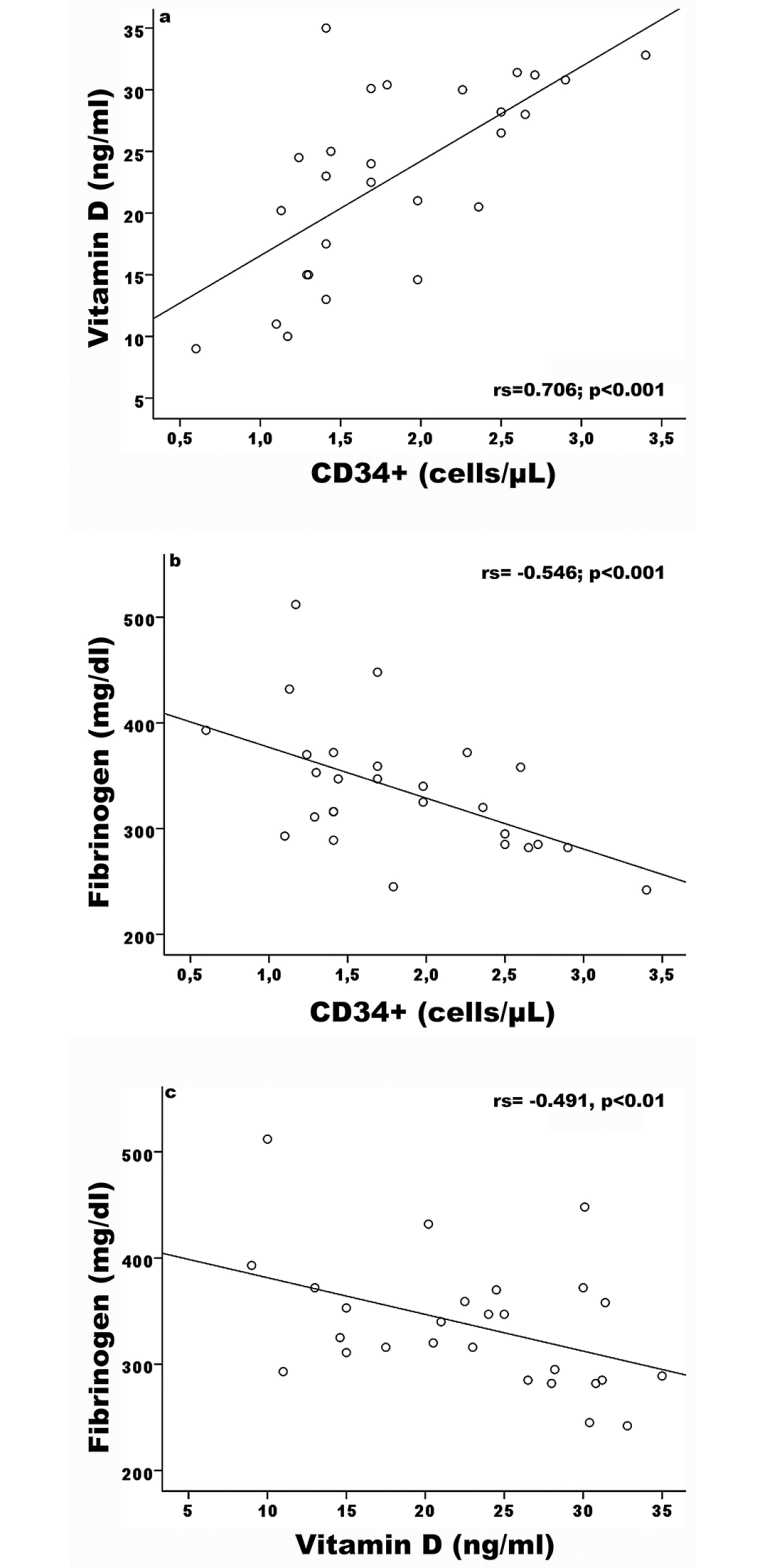
Spearman’s correlation between: CD34+ cell number and Vitamin D levels (a), Fibrinogen (b); Vitamin D levels and Fibrinogen (c). RA subjects. Spearman’s coefficient (rs) is shown with its significance (p).

A correlation was found in RA patients between CRP and PWV (rs = 0.633, p<0.001), but not with cIMT (rs = 0.125, p = 0.84). Correlations among variables are summarized in Table 2. Fig 5 shows the correlations between CD34+ cell number and vitamin D levels (a) and fibrinogen (b), and between vitamin D levels and fibrinogen (c).

**Table 2:** Relationships among the variables.

Whole population	**BMI**	**TC**	**HDL-C**	**CRP**	**Fibrinogen**	**PWV**	**cIMT**	**25-OH D**
**CD34+ cell number**	rs -0.251	rs -0.247	rs -0.112	rs -0.147	rs -0.197	rs -0.302	rs -0.290	rs 0.493
	p = 0.031	p = 0.034	p = ns	p = ns	p = ns	p = 0.017	p = ns	p <0.001
**25-OH D**	rs -0.234	rs -0.126	rs -0.259	rs -0.252	rs -0.371	rs -0.551	rs– 0.408	
	p = 0.045	p = ns	p = 0.026	p = 0.030	p <0.001	p <0.001	p = 0.043	
RA only	**BMI**	**TC**	**HDL-C**	**CRP**	**Fibrinogen**	**PWV**	**cIMT**	**25-OH D**
**CD34+ cell number**	rs -0.151	rs -0.139	rs -0.252	rs -0.092	rs -0.546	rs 0.517	rs 0.451	rs 0.706
	p = ns	p = ns	p = ns	p = ns	P<0.001	p = 0.006	p = 0.018	p <0.001
**25-OH D**	rs -0.166	rs -0.134	rs -0.187	rs -0.203	rs -0.491	rs 0.207	rs 0.326	
	p = ns	p = ns	p = ns	p = ns	p = 0.009	p = ns	p = ns	

Correlations among the variables; upper panel: whole population; lower panel: RA patients only. rs: Spearman’s coefficient; p: level of significance of rs.

In order to assess the contribution of each variable on CD34+ cell number, we performed a multiple linear regression analysis; dependence analysis (Table 3) suggested that the main variable associated to CD34+ cell number was Vitamin D (β = 0.567; B: 0.060, 95%CI: 0.033; 0.087; p <0.001), together with fibrinogen levels (β = -0.323; B: -0.060, 95%CI: -0.010; -0.002; p <0.005); the model estimated for vitamin D assessed as statistical predictors both CD34+ cell count (β = 0.389; B: 3.100, 95%CI: 1.535; 4.665; p <0.001), and fibrinogen (β = -0.368; B: -0.042, 95%CI: -0.064; -0.020; p <0.001).

**Table 3:** Multiple regression analysis.

Dependent variable	Predictors	B	95% CI for B	Beta	T	P
**CD34+ cell number**	25-OH D	0.060	0.033; 0.087	0.567	4.573	<0.001
	Fibrinogen	-0.060	-0.010; -0.002	-0.323	-3.624	<0.005
**25-OH D levels**	CD34+ cells	3.100	1.535; 4.665	0.389	3.949	<0.001
	Fibrinogen	-0.042	-0.064; -0.020	-0.368	-3.736	<0.001

Multiple regression analysis for CD34+ cell number, and 25-OH D levels, in RA subjects. B, unstandardized regression coefficient; 95%CI, 95% Confidence Interval for B; Beta, standardized regression coefficient; T, t-test for Beta; p, p-value for significance.

## Discussion

Considerable evidence indicates that patients with RA have an increased risk of developing CVD [1, 3]. The proinflammatory status occurring in RA is characterized by activation of inflammatory cells, increased levels of inflammatory mediators and the presence of autoantibodies, concurring in inducing endothelial dysfunction, vascular wall stiffening and thickening, and premature atherosclerosis [2, 4]. Endothelial damage can be counteracted by endothelial repair, and circulating progenitor cells originating from the bone marrow may contribute at least in part to this process [5, 10, 37]. Several cell phenotypes have been proposed as EPCs and considered as potential spare cells, able to participate in the turnover of healthy and damaged vascular endothelium; low EPCs number has been shown to correlate with a higher incidence of CV events [9, 38], supporting the notion that EPCs are relevant in the pathophysiology of CVD. Although the question of which cell phenotype better identifies the “true” circulating EPC is to date unsolved [10, 11], by evaluating the impact of different CPC immunophenotypes on the ability to predict or associate with CV risk and outcomes, it has been suggested that CD34+ cell count may be more closely linked to CV risk than CD133+ cell number and multiple positive phenotypes, underlining that a more extensively characterization of circulating CD34+ cells may not be useful for this purpose [14, 15]. The surface antigen CD34, in fact, appears to identify a cluster of cells with progenitor and stem activity, and, in many cases, CD34+ population shows an important differentiation and also transdifferentiation ability [12, 13]. Consistently, CD34+ cell count has been often proposed as prognostic marker in several different clinical settings [5, 6, 9–12, 14, 15, 21].

Indeed, it has been suggested that in RA patients the impairment of endothelial repair mechanisms may be at least in part due to the altered number or function of PHCs; in addition, a novel T cell subset, the so-called angiogenic T cells (Tang), has been more recently suggested to cooperate with circulating progenitors. To date, the relationships between Tang and the different subsets of circulating progenitors are not at all defined, as were their prognostic value in the different clinical settings, but it has been suggested that Tang may be needed to enhance angiogenic properties and endothelial repair function of CD34+ cells, possibly through the secretion of proangiogenic cytokines [16–18].

According with previous studies [21, 22], we confirm a reduction of CD34+ cells in RA patients which might be associated to the chronic inflammatory status. It is known, in fact, that a loss of function and apoptosis of CD34 + cells occur during chronic inflammatory diseases [39]; more recently, it has been also shown a direct link between high α-interferon, low EPC count and increased CV risk [40]. Also, anti-TNFα therapy seems able to restore CPC number in juvenile arthritis [41].

In addition, as already recently reported, we found low levels of Vitamin D in RA patients [27, 42]. In RA patients with moderate disease activity we found an interdependence between CD34+ cell number and early atherosclerosis, as defined by PWV increase and carotid intima-media thickening; CD34+ cell number, also, appears to be affected by low 25-OH D levels.

Vitamin D deficiency is a condition independently associated to adverse CV events [24], and lower serum 25-OH D is associated to inflammation-linked vascular endothelial dysfunction [25] and coronary artery disease, but not to peripheral arterial disease or arterial stiffening [26]. Vitamin D might have a role in the proliferation and differentiation of stem cells, also toward endothelial phenotype, by modulating phospholipase C, VEGF1 and 2, and pro-MMP-2 activity [30, 43]. In addition, it was also proposed that vitamin D might protect PHCs from oxidative stress [44]; in fact, as we previously showed, CD34+ cells from RA patients have increased intracellular ROS levels and imbalanced antioxidant enzymes [22] that could explain at least in part the reduced PHC number found in these patients. Consistently, RA patients with higher vitamin D levels presented with higher CD34+ cell number.

Vitamin D deficiency is commonly associated with inflammatory diseases; this association could be due to an increased prevalence of inflammatory diseases in hypovitaminosis D, although reverse causality cannot be excluded [45]. We also observed a correlation between vitamin D levels and inflammatory markers such as fibrinogen. Reid et al reported that plasma concentrations of 25-OH D decrease after an inflammatory insult, and this decrease appears to persist for several months [46]. However, the relationships with inflammatory markers and the potential role of vitamin D appear to be controversial; in a recent analysis Amer and Qayyum confirmed an inverse relation between vitamin D and CRP at lower levels only, while higher serum levels of vitamin D appear to be associated with higher levels of CRP at least in asymptomatic subjects [47]. In RA it has already been described an association between vitamin D levels and erythrocyte sedimentation rate (ESR), disease activity scores, and also with IL-17 and IL-23 serum levels [48]. Also, Ranganathan et al confirmed an association between vitamin D insufficiency and serum levels of IL-17 in patients with RA [42]. Furthermore, Vitamin D and its analogues inhibit IL-2 and IFN-γ production and stimulates the effects of T-helper type 2 lymphocytes, leading to a reduction in MMPs, and delaying atherosclerotic plaque progression [49]. Vitamin D supplementation has been already proposed as adjuvant treatment for neurological, cardiovascular, respiratory and metabolic diseases, as well in chronic inflammatory diseases; however, the role of vitamin D supplementation in modifying the clinical outcomes has not been definitely confirmed [50–54], and it remains unclear whether vitamin D status is causally related to the pathogenesis of the diseases or is merely a marker of health [55].

We also confirmed an increased carotid AS in RA patients with moderate disease activity, which do not appear to be directly associated to serum vitamin D levels. The role of vitamin D in vascular stiffening is supposed to be multifactorial, and different plausible mechanisms may explain how vitamin D is involved in cardiometabolic outcomes; there is evidence about the implication of vitamin D in regulating renin angiotensin system, proliferation of vascular smooth muscle, insulin resistance, anticoagulant activity, macrophage activation and cytokine generation [23, 24].

Our results, although do not provide a defined mechanism by which vitamin D may interact with CD34+ cells and fibrinogen, suggest that vitamin D could be involved in endothelium homeostasis.

This study however presents several limitations. The first limitation is the small sample size; according to inclusion/exclusion criteria, in fact, we enrolled 27 patients only with moderate disease activity. Second, we investigated the association between inflammation, vitamin D levels and CD34+ cells without a focused investigation on mechanistic and pathophysiological aspects that could explain this association, but this was not the purpose of the study.

In conclusion, our findings confirm Vitamin D deficiency in RA patients, and its association with inflammation; moreover, we suggest that CD34+ cell number is reduced in RA patients with moderate disease activity, mainly in subjects with lower vitamin D levels.

It is likely that this novel association may, at least in part, contribute to explaining the increase of CV morbidity and mortality in patients suffering from RA. Further studies on larger sample sizes could clarify whether a supplementation of Vitamin D could modify CD34+ levels and inflammatory indices in patients affected by rheumatoid arthritis and therefore contributing to reduce cardiovascular risk in this patients.

## References

[1] Meune C, Touze E, Trinquart L, Allanore Y. Trends in cardiovascular mortality in patients with rheumatoid arthritis over 50 years: a systematic review and meta-analysis of cohort studies. Rheumatology (Oxford). 2009;48(10):1309–13. Epub 2009/08/22. 10.1093/rheumatology/kep252 kep252 [pii]. . 19696061

[2] Ku IA, Imboden JB, Hsue PY, Ganz P. Rheumatoid arthritis: model of systemic inflammation driving atherosclerosis. Circ J. 2009;73(6):977–85. Epub 2009/05/12. JST.JSTAGE/circj/CJ-09-0274 [pii]. . 19430165 10.1253/circj.cj-09-0274

[3] Myasoedova E, Chandran A, Ilhan B, Major BT, Michet CJ, Matteson EL, et al The role of rheumatoid arthritis (RA) flare and cumulative burden of RA severity in the risk of cardiovascular disease. Ann Rheum Dis. 2015 Epub 2015/02/01. annrheumdis-2014-206411 [pii]. 10.1136/annrheumdis-2014-206411 . 25637001 PMC4520792

[4] Choy E, Ganeshalingam K, Semb AG, Szekanecz Z, Nurmohamed M. Cardiovascular risk in rheumatoid arthritis: recent advances in the understanding of the pivotal role of inflammation, risk predictors and the impact of treatment. Rheumatology (Oxford). 2014;53(12):2143–54. Epub 2014/06/08. 10.1093/rheumatology/keu224 keu224 [pii]. 24907149 PMC4241890

[5] de Boer HC, Hovens MM, van Oeveren-Rietdijk AM, Snoep JD, de Koning EJ, Tamsma JT, et al Human CD34+/KDR+ cells are generated from circulating CD34+ cells after immobilization on activated platelets. Arterioscler Thromb Vasc Biol. 2011;31(2):408–15. Epub 2010/10/30. ATVBAHA.110.216879 [pii]. 10.1161/ATVBAHA.110.216879 . 21030714

[6] Sidney LE, Branch MJ, Dunphy SE, Dua HS, Hopkinson A. Concise review: evidence for CD34 as a common marker for diverse progenitors. Stem Cells. 2014;32(6):1380–9. Epub 2014/02/06. 10.1002/stem.1661 24497003 PMC4260088

[7] Yoder MC. Endothelial progenitor cell: a blood cell by many other names may serve similar functions. J Mol Med (Berl). 2013;91(3):285–95. Epub 2013/02/02. 10.1007/s00109-013-1002-8 23371317 PMC3704045

[8] Di Stefano R, Felice F, Feriani R, Balbarini A. Endothelial progenitor cells, cardiovascular risk factors and lifestyle modifications. Intern Emerg Med. 2013;8 Suppl 1:S47–9. Epub 2013/03/12. 10.1007/s11739-013-0915-0 . 23475208

[9] Mandraffino G, Sardo MA, Riggio S, D'Ascola A, Alibrandi A, Saitta C, et al Circulating progenitor cells and the elderly: a seven-year observational study. Exp Gerontol. 2012;47(5):394–400. Epub 2012/03/28. 10.1016/j.exger.2012.03.007 S0531-5565(12)00062-9 [pii]. . 22449458

[10] Richardson MR, Yoder MC. Endothelial progenitor cells: quo vadis? J Mol Cell Cardiol. 2011;50(2):266–72. Epub 2010/08/03. 10.1016/j.yjmcc.2010.07.009 S0022-2828(10)00273-7 [pii]. 20673769 PMC3444239

[11] Hirschi KK, Ingram DA, Yoder MC. Assessing identity, phenotype, and fate of endothelial progenitor cells. Arterioscler Thromb Vasc Biol. 2008;28(9):1584–95. Epub 2008/08/02. ATVBAHA.107.155960 [pii]. 10.1161/ATVBAHA.107.155960 . 18669889 PMC5244813

[12] Fadini GP, Losordo D, Dimmeler S. Critical re-evaluation of endothelial progenitor cell phenotypes for therapeutic and diagnostic use. Circulation Research. 2012;110(4):624–37. 10.1161/circresaha.111.243386 PMC3382070. 22343557 PMC3382070

[13] Chao H, Hirschi KK. Hemato-vascular Origins of Endothelial Progenitor Cells? Microvascular research. 2010;79(3):169–73. 10.1016/j.mvr.2010.02.003 PMC2857563. 20149806 PMC2857563

[14] Fadini GP, de Kreutzenberg SV, Coracina A, Baesso I, Agostini C, Tiengo A, et al Circulating CD34+ cells, metabolic syndrome, and cardiovascular risk. Eur Heart J. 2006;27(18):2247–55. Epub 2006/08/17. 10.1093/eurheartj/ehl198 . 16912055

[15] Rigato M, Bittante C, Albiero M, Avogaro A, Fadini GP. Circulating progenitor cell count predicts microvascular outcomes in type 2 diabetic patients. J Clin Endocrinol Metab. 2015:jc20151687. Epub 2015/05/06. 10.1210/jc.2015-1687 . 25942480

[16] Lee JH, Lee SH, Yoo SY, Asahara T, Kwon SM. CD34 hybrid cells promote endothelial colony-forming cell bioactivity and therapeutic potential for ischemic diseases. Arterioscler Thromb Vasc Biol. 2013;33(7):1622–34. Epub 2013/05/04. 10.1161/ATVBAHA.112.301052 ATVBAHA.112.301052 [pii]. . 23640491

[17] Rodriguez-Carrio J, Alperi-Lopez M, Lopez P, Alonso-Castro S, Ballina-Garcia FJ, Suarez A. Angiogenic T cells are decreased in rheumatoid arthritis patients. Ann Rheum Dis. 2015;74(5):921–7. Epub 2014/01/09. 10.1136/annrheumdis-2013-204250 annrheumdis-2013-204250 [pii]. . 24399233

[18] Hur J, Yang HM, Yoon CH, Lee CS, Park KW, Kim JH, et al Identification of a novel role of T cells in postnatal vasculogenesis: characterization of endothelial progenitor cell colonies. Circulation. 2007;116(15):1671–82. Epub 2007/10/03. CIRCULATIONAHA.107.694778 [pii]. 10.1161/CIRCULATIONAHA.107.694778 . 17909106

[19] Park YJ, Kim JY, Park J, Choi JJ, Kim WU, Cho CS. Bone erosion is associated with reduction of circulating endothelial progenitor cells and endothelial dysfunction in rheumatoid arthritis. Arthritis Rheumatol. 2014;66(6):1450–60. Epub 2014/07/06. . 24991663 10.1002/art.38352

[20] Herbrig K, Haensel S, Oelschlaegel U, Pistrosch F, Foerster S, Passauer J. Endothelial dysfunction in patients with rheumatoid arthritis is associated with a reduced number and impaired function of endothelial progenitor cells. Ann Rheum Dis. 2006;65(2):157–63. Epub 2005/06/25. ard.2005.035378 [pii]. 10.1136/ard.2005.035378 15975971 PMC1798039

[21] Lo Gullo A, Mandraffino G, Imbalzano E, Mamone F, Aragona CO, D'Ascola A, et al Toll-like receptor 3 and interleukin 1beta expression in CD34+ cells from patients with rheumatoid arthritis: association with inflammation and vascular involvement. Clin Exp Rheumatol. 2014;32(6):922–9. Epub 2014/12/02. 8062 [pii]. . 25436985

[22] Lo Gullo A, Mandraffino G, Sardo M, D'Ascola A, Mamone F, Loddo S, et al Circulating progenitor cells in rheumatoid arthritis: association with inflammation and oxidative stress. Scand J Rheumatol. 2013 Epub 2013/12/10. 10.3109/03009742.2013.836564 . 24313545

[23] Pittas AG, Chung M, Trikalinos T, Mitri J, Brendel M, Patel K, et al Systematic review: Vitamin D and cardiometabolic outcomes. Ann Intern Med. 2010;152(5):307–14. Epub 2010/03/03. 10.7326/0003-4819-152-5-201003020-00009 152/5/307 [pii]. 20194237 PMC3211092

[24] Lavie CJ, Lee JH, Milani RV. Vitamin D and cardiovascular disease will it live up to its hype? J Am Coll Cardiol. 2011;58(15):1547–56. Epub 2011/10/01. 10.1016/j.jacc.2011.07.008 S0735-1097(11)02648-9 [pii]. . 21958881

[25] Jablonski KL, Chonchol M, Pierce GL, Walker AE, Seals DR. 25-Hydroxyvitamin D deficiency is associated with inflammation-linked vascular endothelial dysfunction in middle-aged and older adults. Hypertension. 2011;57(1):63–9. Epub 2010/12/01. 10.1161/HYPERTENSIONAHA.110.160929 21115878 PMC3020150

[26] Liew JY, Sasha SR, Ngu PJ, Warren JL, Wark J, Dart AM, et al Circulating vitamin D levels are associated with the presence and severity of coronary artery disease but not peripheral arterial disease in patients undergoing coronary angiography. Nutr Metab Cardiovasc Dis. 2014 Epub 2015/02/03. S0939-4753(14)00370-6 [pii]. 10.1016/j.numecd.2014.12.005 . 25640800

[27] Rossini M, Maddali Bongi S, La Montagna G, Minisola G, Malavolta N, Bernini L, et al Vitamin D deficiency in rheumatoid arthritis: prevalence, determinants and associations with disease activity and disability. Arthritis Res Ther. 2010;12(6):R216 Epub 2010/12/01. 10.1186/ar3195 ar3195 [pii]. 21114806 PMC3046526

[28] Haque UJ, Bathon JM, Giles JT. Association of vitamin D with cardiometabolic risk factors in rheumatoid arthritis. Arthritis Care Res (Hoboken). 2012;64(10):1497–504. Epub 2012/05/05. 10.1002/acr.21715 22555877 PMC3462271

[29] Cianciolo G, La Manna G, Cappuccilli ML, Lanci N, Della Bella E, Cuna V, et al VDR expression on circulating endothelial progenitor cells in dialysis patients is modulated by 25(OH)D serum levels and calcitriol therapy. Blood Purif. 2011;32(3):161–73. Epub 2011/07/16. 10.1159/000325459 000325459 [pii]. . 21757895

[30] Grundmann M, Haidar M, Placzko S, Niendorf R, Darashchonak N, Hubel CA, et al Vitamin D improves the angiogenic properties of endothelial progenitor cells. Am J Physiol Cell Physiol. 2012;303(9):C954–62. Epub 2012/08/31. 10.1152/ajpcell.00030.2012 ajpcell.00030.2012 [pii]. 22932684 PMC3492823

[31] Arnett FC, Edworthy SM, Bloch DA, McShane DJ, Fries JF, Cooper NS, et al The American Rheumatism Association 1987 revised criteria for the classification of rheumatoid arthritis. Arthritis Rheum. 1988;31(3):315–24. Epub 1988/03/01. . 3358796 10.1002/art.1780310302

[32] Ross AC, Manson JE, Abrams SA, Aloia JF, Brannon PM, Clinton SK, et al The 2011 Dietary Reference Intakes for Calcium and Vitamin D: what dietetics practitioners need to know. J Am Diet Assoc. 2011;111(4):524–7. Epub 2011/03/30. 10.1016/j.jada.2011.01.004 S0002-8223(11)00005-8 [pii]. . 21443983

[33] Mandraffino G, Sardo MA, Riggio S, Loddo S, Imbalzano E, Alibrandi A, et al Circulating progenitor cells are increased in newly diagnosed untreated hypertensive patients with arterial stiffening but normal carotid intima-media thickness. Hypertens Res. 2011;34(7):876–83. Epub 2011/05/20. 10.1038/hr.2011.56 hr201156 [pii]. . 21593737

[34] Barnett D, Janossy G, Lubenko A, Matutes E, Newland A, Reilly JT. Guideline for the flow cytometric enumeration of CD34+ haematopoietic stem cells. Prepared by the CD34+ haematopoietic stem cell working party. General Haematology Task Force of the British Committee for Standards in Haematology. Clin Lab Haematol. 1999;21(5):301–8. Epub 2000/01/26. . 10.1046/j.1365-2257.1999.00253.x 10646072

[35] Mancia G, De Backer G, Dominiczak A, Cifkova R, Fagard R, Germano G, et al 2007 ESH-ESC Practice Guidelines for the Management of Arterial Hypertension: ESH-ESC Task Force on the Management of Arterial Hypertension. J Hypertens. 2007;25(9):1751–62. Epub 2007/09/01. 10.1097/HJH.0b013e3282f0580f 00004872-200709000-00001 [pii]. . 17762635

[36] Pesarin F, Salmaso L. Permutation Tests for Complex Data: Theory, Applications and Software: Wiley; 2010.

[37] Steinmetz M, Nickenig G, Werner N. Endothelial-regenerating cells: an expanding universe. Hypertension. 2010;55(3):593–9. Epub 2010/01/20. HYPERTENSIONAHA.109.134213 [pii]. 10.1161/HYPERTENSIONAHA.109.134213 . 20083733

[38] Hill JM, Zalos G, Halcox JP, Schenke WH, Waclawiw MA, Quyyumi AA, et al Circulating endothelial progenitor cells, vascular function, and cardiovascular risk. N Engl J Med. 2003;348(7):593–600. Epub 2003/02/14. 10.1056/NEJMoa022287 348/7/593 [pii]. . 12584367

[39] Verma S, Kuliszewski MA, Li SH, Szmitko PE, Zucco L, Wang CH, et al C-reactive protein attenuates endothelial progenitor cell survival, differentiation, and function: further evidence of a mechanistic link between C-reactive protein and cardiovascular disease. Circulation. 2004;109(17):2058–67. Epub 2004/04/14. 10.1161/01.CIR.0000127577.63323.24 01.CIR.0000127577.63323.24 [pii]. . 15078802

[40] Rodriguez-Carrio J, de Paz B, Lopez P, Prado C, Alperi-Lopez M, Ballina-Garcia FJ, et al IFNalpha serum levels are associated with endothelial progenitor cells imbalance and disease features in rheumatoid arthritis patients. PLoS One. 2014;9(1):e86069 Epub 2014/01/28. 10.1371/journal.pone.0086069 PONE-D-13-38115 [pii]. 24465874 PMC3897639

[41] Martini G, Biscaro F, Boscaro E, Calabrese F, Lunardi F, Facco M, et al Reduced levels of circulating progenitor cells in juvenile idiopathic arthritis are counteracted by anti TNF-alpha therapy. BMC Musculoskelet Disord. 2015;16(1):103 Epub 2015/05/01. 10.1186/s12891-015-0555-9 10.1186/s12891-015-0555-9 [pii]. 25925313 PMC4418050

[42] Ranganathan P, Khalatbari S, Yalavarthi S, Marder W, Brook R, Kaplan MJ. Vitamin D deficiency, interleukin 17, and vascular function in rheumatoid arthritis. J Rheumatol. 2013;40(9):1529–34. Epub 2013/07/03. 10.3899/jrheum.130012 jrheum.130012 [pii]. . 23818717 PMC4358878

[43] Bickford PC, Tan J, Shytle RD, Sanberg CD, El-Badri N, Sanberg PR. Nutraceuticals synergistically promote proliferation of human stem cells. Stem Cells Dev. 2006;15(1):118–23. Epub 2006/03/09. 10.1089/scd.2006.15.118 . 16522169

[44] Uberti F, Lattuada D, Morsanuto V, Nava U, Bolis G, Vacca G, et al Vitamin D protects human endothelial cells from oxidative stress through the autophagic and survival pathways. J Clin Endocrinol Metab. 2014;99(4):1367–74. Epub 2013/11/29. 10.1210/jc.2013-2103 jc.2013-2103 [pii]. . 24285680

[45] Silva MC, Furlanetto TW. Does serum 25-hydroxyvitamin D decrease during acute-phase response? A systematic review. Nutr Res. 2015;35(2):91–6. Epub 2015/01/30. 10.1016/j.nutres.2014.12.008 . 25631715

[46] Reid D, Toole BJ, Knox S, Talwar D, Harten J, O'Reilly DS, et al The relation between acute changes in the systemic inflammatory response and plasma 25-hydroxyvitamin D concentrations after elective knee arthroplasty. Am J Clin Nutr. 2011;93(5):1006–11. Epub 2011/03/18. 10.3945/ajcn.110.008490 ajcn.110.008490 [pii]. . 21411617

[47] Amer M, Qayyum R. Relation between serum 25-hydroxyvitamin D and C-reactive protein in asymptomatic adults (from the continuous National Health and Nutrition Examination Survey 2001 to 2006). Am J Cardiol. 2012;109(2):226–30. Epub 2011/10/15. 10.1016/j.amjcard.2011.08.032 S0002-9149(11)02748-2 [pii]. . 21996139

[48] Hong Q, Xu J, Xu S, Lian L, Zhang M, Ding C. Associations between serum 25-hydroxyvitamin D and disease activity, inflammatory cytokines and bone loss in patients with rheumatoid arthritis. Rheumatology (Oxford). 2014;53(11):1994–2001. Epub 2014/06/08. 10.1093/rheumatology/keu173 keu173 [pii]. . 24907153

[49] Andress DL. Vitamin D in chronic kidney disease: a systemic role for selective vitamin D receptor activation. Kidney Int. 2006;69(1):33–43. Epub 2005/12/24. 5000045 [pii] 10.1038/sj.ki.5000045 . 16374421

[50] Bjelakovic G, Gluud LL, Nikolova D, Whitfield K, Wetterslev J, Simonetti RG, et al Vitamin D supplementation for prevention of mortality in adults. Cochrane Database Syst Rev. 2014;1:CD007470 Epub 2014/01/15. 10.1002/14651858.CD007470.pub3 . 24414552 PMC11285307

[51] Beveridge LA, Struthers AD, Khan F, Jorde R, Scragg R, Macdonald HM, et al Effect of Vitamin D Supplementation on Blood Pressure: A Systematic Review and Meta-analysis Incorporating Individual Patient Data. JAMA Intern Med. 2015 Epub 2015/03/17. 10.1001/jamainternmed.2015.0237 2195120 [pii]. . 25775274 PMC5966296

[52] Antico A, Tampoia M, Tozzoli R, Bizzaro N. Can supplementation with vitamin D reduce the risk or modify the course of autoimmune diseases? A systematic review of the literature. Autoimmun Rev. 2012;12(2):127–36. Epub 2012/07/11. 10.1016/j.autrev.2012.07.007 . 22776787

[53] Seida JC, Mitri J, Colmers IN, Majumdar SR, Davidson MB, Edwards AL, et al Clinical review: Effect of vitamin D3 supplementation on improving glucose homeostasis and preventing diabetes: a systematic review and meta-analysis. J Clin Endocrinol Metab. 2014;99(10):3551–60. Epub 2014/07/26. 10.1210/jc.2014-2136 . 25062463 PMC4483466

[54] Dalbeni A, Scaturro G, Degan M, Minuz P, Delva P. Effects of six months of vitamin D supplementation in patients with heart failure: a randomized double-blind controlled trial. Nutr Metab Cardiovasc Dis. 2014;24(8):861–8. Epub 2014/05/03. 10.1016/j.numecd.2014.02.015 S0939-4753(14)00086-6 [pii]. . 24787908

[55] Ponda MP, Huang X, Odeh MA, Breslow JL, Kaufman HW. Vitamin D may not improve lipid levels: a serial clinical laboratory data study. Circulation. 2012;126(3):270–7. Epub 2012/06/22. 10.1161/circulationaha.111.077875 22718799 PMC3713625

